# Ventricular Arrhythmias Due to Glomangiosarcoma Cardiac Metastases

**DOI:** 10.1016/j.jaccao.2020.11.015

**Published:** 2021-03-16

**Authors:** Chiara Lestuzzi, Giammaria Miolo, Lucia Tartuferi, Rosa Pecoraro, Antonino De Paoli, Ermanno Dametto, Nadir Sitta, Elisa Leiballi, Fabio D. Greco, Angela Buonadonna

**Affiliations:** a Cardio-Oncology Service, Department of Cardiology, Azienda Sanitaria Friuli Occidentale, Centro di Riferimento Oncologico di Aviano, Istituto di Ricovero e Cura a Carattere Scientifico, Aviano, Italy; b Department of Medical Oncology, National Cancer Institute, Centro di Riferimento Oncologico di Aviano, Istituto di Ricovero e Cura a Carattere Scientifico, Aviano, Italy; c Department of Cardiology, Azienda Sanitaria Friuli Occidentale, Pordenone, Italy; d Department of Radiation Oncology, National Cancer Institute, Centro di Riferimento Oncologico di Aviano, Istituto di Ricovero e Cura a Carattere Scientifico, Aviano, Italy; e Department of Cardiology, Unità Locale Socio-Sanitaria (ULSS)2, Conegliano, Italy; f Department of Radiology, Azienda Sanitaria Friuli Occidentale, Pordenone, Italy

**Keywords:** antiangiogenic therapy, arrhythmia, cancer survivorship, cardiac magnetic resonance, cardiac masses, outcomes, palliative care, sarcoma, treatment, tyrosine kinase inhibitor

**Figure:**
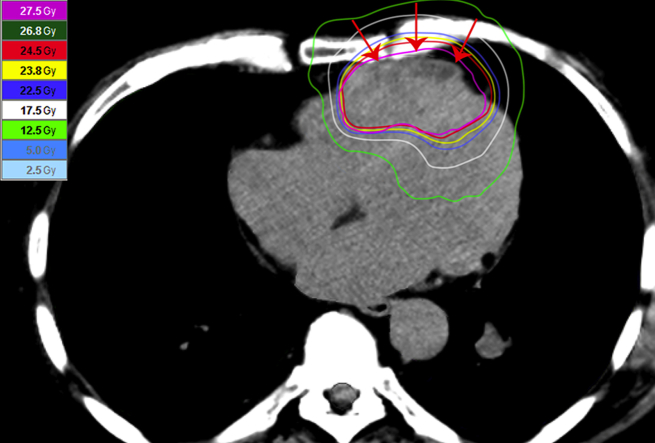

In December 2013, a 61-year-old man diagnosed with glomangiosarcoma of the right subclavian artery with an intermediate grade of malignancy (G2) was treated with neoadjuvant chemotherapy including epidoxorubicin and ifosfamide, and radiotherapy, followed by radical surgery in June 2014. In October 2014, computed tomography (CT) detected bilateral pulmonary metastases and a mass within the right ventricular wall. Echocardiography and cardiac magnetic resonance showed 2 confluent masses within the right ventricular free wall (4 × 1.5 cm) and an apical mass (2.5 × 1.5 cm). Treatment with nonpegylated liposomal doxorubicin and ifosfamide prevented disease progression for 1 year. In February 2016, positron emission tomography (PET)-CT showed pulmonary progression. Several lines of chemotherapy (gemcitabine, trabectedin, dacarbazine, paclitaxel) were attempted, but each was interrupted due to systemic toxicity or pulmonary metastases progression. In April 2017, when imaging revealed further lung progression and an increase in the dimensions of the cardiac mass, dacarbazine chemotherapy was restarted.

One week later, the patient was seen in the cardiology clinic for dizziness and concerns over an abnormal heart rate. Electrocardiography revealed normal sinus rhythm with frequent ventricular ectopic beats (VEBs) originating from the right ventricle; 24-h Holter monitoring recorded 1,069 VEBs (1.1% of all beats), with 72 couplets and 133 runs of nonsustained ventricular tachycardia (NSVT) (Figure 1). Metoprolol 50 mg twice daily was prescribed, but after 1 week, the patient continued to experience dizziness, with 2,405 VEBs (2.7% of beats), 194 couplets, and 188 runs of NSVT detected on 24-h Holter monitoring. After changing his regimen to amiodarone 200 mg daily, the patient’s symptoms quickly improved, and the VEBs ceased.

**Figure 1:**
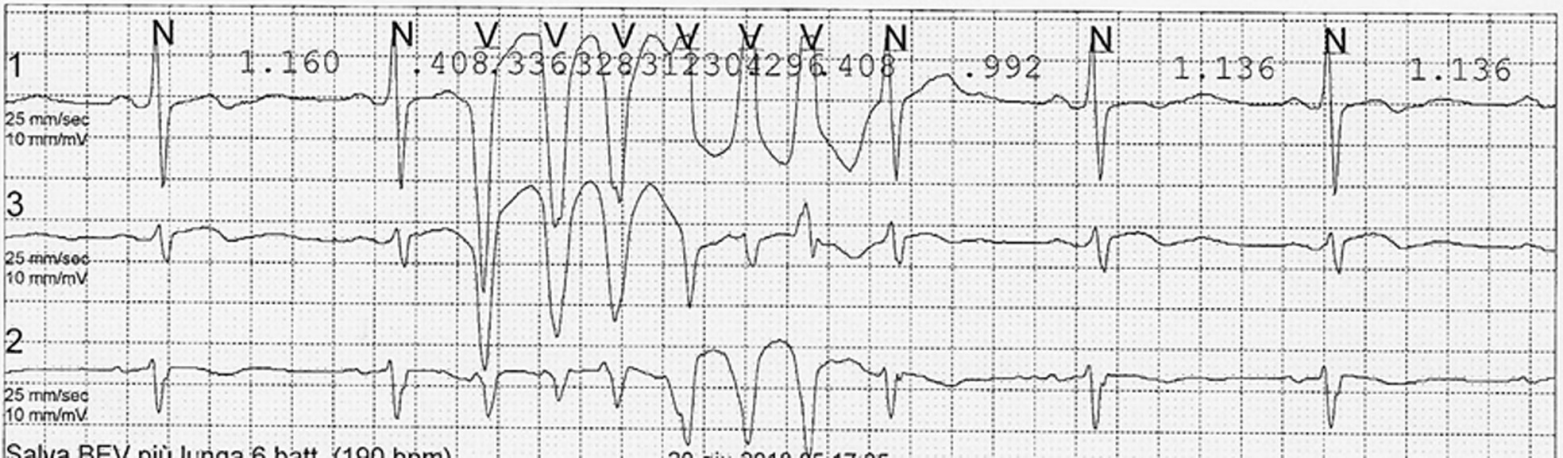
Holter Monitoring in May 2017 Holter monitor recording showing polymorphic ventricular tachycardia.

At oncologic restaging in July 2017, CT showed pulmonary progression, requiring a change in therapy. The only appropriate drug candidate was pazopanib, a tyrosine kinase inhibitor used in treatment of advanced soft tissue sarcomas and recently indicated in angiosarcoma (1). Given the interaction between amiodarone and pazopanib metabolism, as both drugs prolong the QT interval and affect thyroid function (2,3), amiodarone was discontinued, and, after a 2-week washout, pazopanib 800 mg daily was started in August 2017, with a plan for frequent electrocardiogram and Holter monitoring. Only rare, isolated VEBs were observed for more than 1 month after starting pazopanib.

However, beginning in mid-September, systemic toxicities developed, including nausea, vomiting, and fatigue, resulting in the patient not taking his prescribed dose regularly. Holter monitoring revealed frequent VEBs, few couplets, and rare triplets. Pazopanib was thus discontinued in October 2017 in anticipation of restaging 1 month later. During the period of discontinuation, isolated VEBs and couplets increased significantly (up to 17% of total beats), and episodes of VT were more frequent (up to 72/day) and more prolonged. Because the patient was not experiencing significant clinical symptoms, however, antiarrhythmic treatment was not prescribed.

In November 2017, a restaging CT showed a slight reduction in the lung lesions’ dimension, without changes in the heart metastases. Pazopanib was therefore restarted at a reduced dose of 600 mg daily, resulting in a reduction of ventricular arrhythmias (VAs) to fewer than 30 isolated VEBs/h. However, the patient again self-decreased his pazopanib therapy in the following months due to systemic toxicities. In January 2018, after 4 days without pazopanib, Holter monitoring recorded 9,610 VEBs with 173 episodes of VT. This worsened in March 2018, when after 1 week without pazopanib, Holter monitoring recorded frequent polymorphic VEBs with 1,006 couplets and 731 runs (fastest at 226 beats/min). Bisoprolol 5 mg daily was added, without significant benefit.

In March 2018, amiodarone 200 mg every other day to daily was restarted. With the addition of amiodarone and pazopanib, the number of VEBs decreased. In May 2018, progression of cardiac metastases was observed, as was worsening of VEBs. A progressive increase in amiodarone dose (up to 5 tablets/week) was initiated. By the end of June, the patient’s general clinical conditions improved, and he was able to resume a low dose of pazopanib that was gradually increased. As Holter monitoring showed a progressive reduction of VEBs, amiodarone dose was reduced and then stopped in January 2019, when no VEBs were recorded.

Until the end of March 2019, the patient was compliant with full-dose pazopanib, was not taking any antiarrhythmic therapy, and was not experiencing VA. Pazopanib was discontinued from April to May 2019 to allow surgical removal of a right shoulder relapse. Then, at the end of May, the patient complained again of dizziness, and frequent VEBs with couplets were observed. Amiodarone was restarted. In the following 2 months, there was partial regression of the cardiac metastases, and the number and complexity of VAs progressively declined (from 11.9% to 2.5% of all beats).

We reviewed the 40 Holter monitoring recordings that were taken between August 2017 and July 2019, comparing the VA with and without pazopanib and amiodarone therapy. When the patient was regularly taking pazopanib without amiodarone, the number of VEBs were between 0% and 5% of the total beats. When pazopanib was reduced to 30% to 50% of the prescribed dose, the number of VEBs increased to 10% to 13% of total beats, with 500 to 1,000 couplets and >100 episodes of VT. With the addition of amiodarone (2 to 4 tablets/week), VT fell to <2%. When pazopanib was discontinued, the VEBs increased to 15% to 17%, with up to 2,000 couplets and up to 350 episodes of VT, and amiodarone 200 mg daily was necessary to reduce the VEBs to 4% to 5%. For the most part, the dose of amiodarone required to keep the VEBs <5% was kept inversely proportional to the dose of pazopanib.

In August 2019, VAs increased again despite full doses of pazopanib and amiodarone. PET detected a progression in the dimensions and uptake of both the pulmonary and cardiac metastases. The VAs progressively increased in number, and we increased the amiodarone dose to 400 mg daily. VEBs were 30.7% of all beats, up to >1,000/h. Pazopanib was no longer effective and therefore discontinued. We planned palliative radiotherapy of the cardiac metastases, trying to block the arrhythmic focus. The patient subsequently underwent stereotactic external beam radiotherapy with a dose of 25 Gy in 5 daily fractions. The gross tumor volume (metastases) was included in the isodose of 110% (i.e., 27.5 Gy) (Figure 2). During radiotherapy, VAs worsened, and symptomatic sustained VT occurred (up to 1,179 beats). The patient was admitted to the intensive care unit and treated with magnesium sulfate, methylprednisolone (40 mg/day for 4 days), and high-dose amiodarone in continuous infusion until the end of radiotherapy. The patient was discharged 1 week later on oral amiodarone (400 mg daily) and prednisone (25 mg daily).

**Figure 2:**
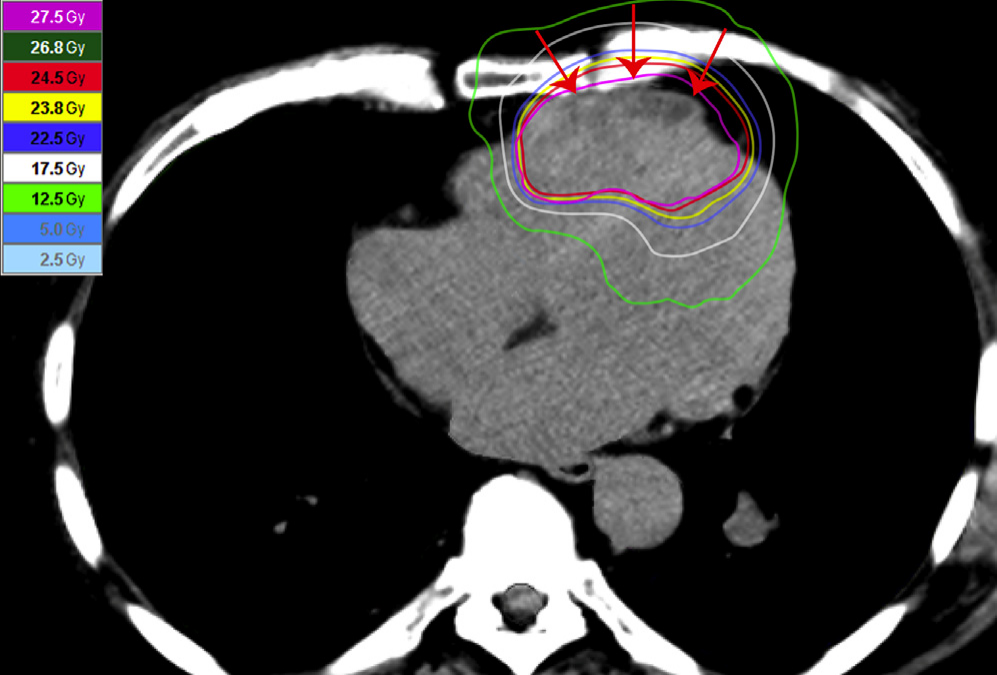
Computed Tomography and Radiotherapy Plan The masses **(arrows)** infiltrate the free wall and the apex of the right ventricle. The **colored lines** contour the intensity-modulated radiation dose (see scale on the **left**). The planned volume takes into account the cardiac motion to keep the metastases within the radiation field throughout the cardiac cycle.

During the following weeks, the episodes of VT decreased and were shorter and slower. At the last follow-up, in December 2019, the patient had cough, hemoptysis, dyspnea, and some palpitations but was not experiencing dizziness. Echocardiography showed a decreased echogenicity of the infiltrating masses (consistent with tumor necrosis), the left ventricular ejection fraction was 66%, and the right ventricular shortening fraction was 50%. On Holter monitoring, there were 11,152 VEBs (13.3% of all beats), 366 couplets, and 152 runs of NSVT (up to 40 beats). The patient subsequently died of pulmonary progression, without arrhythmia-related symptoms.

## Discussion

Glomus tumors are extremely rare neoplasms that usually originate from the glomus body. They are usually benign, but in exceedingly rare cases, they may have more aggressive biologic features (i.e., malignant glomus tumor or glomangiosarcoma). They may be locally infiltrative, slow-growing, relatively large malignancies or, in extremely rare cases, may rapidly metastasize to distant locations, typically leading to mortality, and as such are considered a rare subtype of angiosarcoma (4).

Cardiac metastases from angiosarcoma of other organs have been reported in the medical literature only twice (4,5). At diagnosis, treatment is directed toward reducing tumor burden at metastatic sites—in this case, the lungs and heart—in an effort to enhance the patient’s quality and length of life. Several lines of systemic chemotherapy were attempted and discontinued due to toxicity and pulmonary metastatic progression. Once the patient developed symptoms associated with cardiac metastasis, the goals of care were to effectively suppress ventricular arrhythmia, and with disease progression, palliate symptoms.

In this case, the only clinical sign of myocardial metastases was VA, described in other cardiac tumors (6). The behavior of arrhythmias in this patient was characterized by the appearance after metastatic progression, rapid response to pazopanib treatment, relapse after pazopanib reduction or discontinuation, and progressive worsening when the tumor was no longer responsive and PET demonstrating an increase in metabolic activity. Such characteristics suggest an arrhythmogenic effect of the metastases due not only to changes in size but also in their metabolic activity and may be considered as a disease marker.

The management of symptomatic, malignant VAs was challenging in this case. Beta-blockers were not effective, while amiodarone was, although its concurrent use with pazopanib is not usually recommended. When the tumor no longer responded to pazopanib and the arrhythmias were not controlled by amiodarone, the challenge was to find an acceptable means to control symptoms. We excluded an implantable cardioverter-defibrillator device because it may have worsened the patient’s quality of life by delivering multiple shocks. Radiofrequency ablation (7) and surgery were deemed impossible due to the presence of multiple cardiac lesions. Stereotactic radiotherapy has been successfully employed in selected patients with refractory VA and also had the potential added benefit of reducing tumor burden (8). During radiotherapy, the number and severity of VAs increased, possibly owing to an inflammatory effect, as already described (8,9). Treatment with steroids and high-dose amiodarone were used to support the patient during this critical phase, and the goal of controlling symptoms in this patient was achieved. Highly conformal stereotactic radiotherapy was effective in reducing the cardiac metastases and the related arrhythmias, without causing ventricular dysfunction.

Patients with metastatic angiosarcoma usually have a survival rate of a few months (10), but our patient lived for 5 years following the initial detection of cardiac and lung metastases. The most effective antineoplastic therapy was pazopanib, which has activity against several proangiogenic growth factors overexpressed in the often hypervascular angiosarcomas (1,10), and provided control of the tumor and the VA secondary to myocardial metastases. In the late stage of disease, palliative stereotactic radiotherapy was successful in controlling recurrent VAs.

## Conclusions

In this rare case of cardiac metastases from glomangiosarcoma, intramural metastases caused severe VA, which was a disease marker in this particular patient, and treatment with several therapies, primarily pazopanib, enabled prolonged survival, supporting the use of pazopanib in selected patients. Stereotactic radiotherapy may be considered in cases of refractory or life-threatening VA induced by cardiac tumors, but it may cause a transient worsening of VA.

## Funding Support and Author Disclosures

The authors have reported that they have no relationships relevant to the contents of this paper to disclose.
